# Economic Impact of Abortions in Dairy Cow Herds

**DOI:** 10.3390/vetsci12070645

**Published:** 2025-07-07

**Authors:** Osvaldo Palma, Lluís M. Plà-Aragonès, Alejandro Mac Cawley, Víctor M. Albornoz

**Affiliations:** 1 Department of Mathematics, Universidad de Lleida, 73 Jaume II, 25001 Lleida, Spain; osvaldo.palma@unab.cl; 2 Department of Economics and Administration, Universidad Nacional Andrés Bello, Santiago 7500000, Chile; 3 Agrotecnio CERCA Center, 191, Rovira Roure, 25198 Lleida, Spain; 4 Department of Industrial and Systems Engineering, Pontificia Universidad Católica de Chile, Santiago 7820436, Chile; amac@uc.cl; 5 Department of Industrial Engineering, Campus Santiago Vitacura, Universidad Técnica Federico Santa María, Santiago 7650568, Chile; victor.albornoz@usm.cl

**Keywords:** abortion, replacement, dairy, milk, Markov chain, optimization

## Abstract

This study aimed to explore Markov decision methods in order to solve the problem of dairy cow replacement, adding the special characteristics of two types of abortions due to different sanitary reasons that influence the economic, production, and reproduction performance of these animals. The model was successfully validated against other models published in the literature. Python code v.3.13 was used to solve the problem and to ease future extensions with the inclusion of new variables. The results constitute tools that allow the veterinarian to explore more realistic scenarios by running a Markov simulation model that avoids the complexities leading to the problem of dimensionality in dynamic optimization models. In our study, the economic value of the herd considering RA and NLA abortions shows that the maximum net benefit is USD 178.77 per cow, and non-pregnant cows are slaughtered upon reaching six months of lactation, a value that is within the range of values reported by the literature that we have identified. At the optimum, the replacement model extended with abortion generates a difference of USD 0.69 per cow per month compared to the model that does not include the special abortion features. The changes in the net present value of each cow according to the month of culling depend on the variability of milk income and slaughter value and heifers’ replacement values, suggesting that any measure that seeks to improve the economic benefit of dairy cows should take greater account of these variables.

## Simple Summary

In this study, we address the economic impact of abortions in dairy cow herds using a cow replacement model. We employ a Markov decision model to optimize replacement decisions, considering two types of abortions that affect cow production and reproduction. Our goal was to find the best strategy to maximize the economic benefits of the herd. We found that the greatest benefit is realized when cows that are not pregnant are culled at six months of lactation, resulting in a benefit of USD 178.77 per cow per month. This model helps veterinarians and farmers make informed decisions regarding the replacement of aborted cows, thus improving efficiency and profitability in the long run. The results of this study are valuable because they help farmers manage dairy herds better, reduce costs, and increase their profits.

## 1. Introduction

Many approaches to optimize dairy cow performance have been proposed based on solving the dairy cow replacement problem through Markov decision processes (MDPs) [1] or relying on Markov models (MMs) simulation [2]. A dairy cow herd is a dynamic system, i.e., a population of cows aging while producing milk and are replaced as soon as they stop being profitable. For that reason, the replacement problem is the most important in herd management from the economic point of view, as agreed by many authors [2,3]. Kalantari et al. [4], among others, indicate that the most important factors influencing replacement decisions are the milk yield, pregnancy rate, health status, stage of lactation, and the cost of heifers. Dairy cow replacement policies have a significant influence on the financial performance of the herd [5], and keeping low-performance cows or selecting the wrong moment to replace them implies economic losses or inefficiencies [6]. Additionally, from the mathematical point of view, the problem is complex and difficult to solve [7].

Modeling complexity relies on the uncertainty of herd dynamics and milk production over time, and the variability observed from cow to cow [8]. For example, it is difficult to predict the impact on milk production if a cow has health problems, or the variability in reproduction performance, mortality rate, etc. On the other hand, solving difficulties relies on methods such as Markov decision processes and dynamic programming, reporting the “curse of dimensionality” problem [1,9] associated with computational issues. This is because the inclusion of new features in herd models implies an exponential growth of the state space representation, which makes the number of variables impractical and useless for practitioners and farmers. Cabrera [10] reported a low level of real-life application of the models developed until 2010, and few changes have been observed since then. Additionally, Heikkilä et al. [11] indicated that few herd models explored diseases or reproduction disorders like abortion affecting cow replacement, concluding that they cannot be ignored and encouraging proposals combining replacement models with reproductive failures. For instance, many published models consider abortion risk as one of the causes of reproductive failure, but none of them analyze their impact [6,12,13,14]. It is agreed that large and detailed models can [13] considerably complicate the computation of the optimal solution, leading to model simplifications or to proposed submodels according to the main objective or decision criteria [6]. Monti et al. [15] highlight that voluntary culling, i.e., slaughtering decision, is important for the herd dynamics and must be studied jointly with economics and management practices.

Therefore, studying abortions in dairy cattle is crucial because they significantly affect the profitability of dairy farms [16]. Abortions, especially those occurring late in gestation, result in considerable economic losses due to reduced milk production, increased calving intervals, and additional cow replacement costs. Additionally, abortions can lead to an increase in cow discard time and increased replacement costs for farmers. The main types of abortions include infectious abortions caused by pathogens such as bacteria, viruses, and parasites, and non-infectious abortions resulting from genetic, nutritional, environmental, or management factors. Among non-infectious abortions, abortions stand out: new lactation abortion (NLA) and rebreeding abortion (RA). NLA represents abortions losing the calf but cows producing milk, and so, leading to a new lactation after abortion with different economic and management consequences. RA represents abortions, losing the calf, and cows do not produce milk; therefore, no change in the number of lactations occurs, and the cow is reinseminated to attempt a new pregnancy. Understanding these types and their specific causes allows veterinarians to propose more effective management strategies to mitigate the negative impact of abortions and improve overall herd health. Only one observational study has been found in the literature devoted to value productive and reproductive losses caused by abortion [17]. In our study, we propose Markov decision processes (MDPs) to model replacement decisions in a probabilistic environment and the impact on economic herd performance when we take into account the different types of abortion that a dairy cow can suffer.

## 2. Materials and Methods

**Figure 1:**
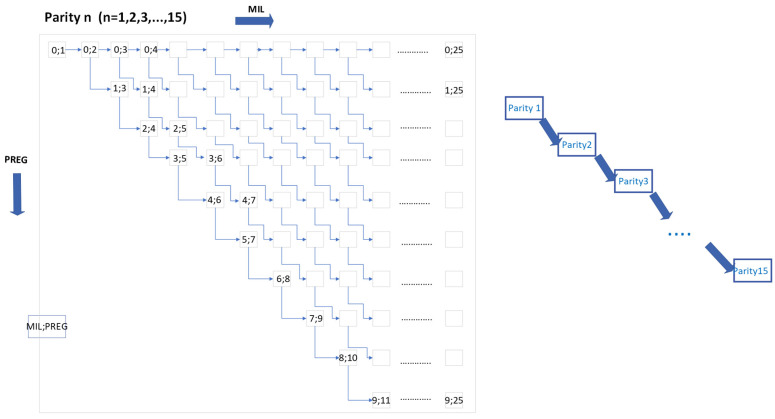
Representation of feasible transitions. Each cow starts at PREG = 0, MIL = 1, and PAR = 1. Each state has associated probabilities of abortion, mortality, and involuntary culling. States with PREG = 0 and MIL = m have an associated probability of becoming pregnant, moving to the state PREG = 1 and MIL = m + 1; otherwise, the cow goes to the state PREG = 0 and MIL = m + 1.

An MDP is a particular stochastic optimization model that describes a system by states and actions that affect the transitions between these states in a probabilistic way [18]. Each action has a reward associated with it, and the goal is to find an optimal policy that maximizes the reward accrued over time. Key components include states, actions, transitions, rewards, and the optimal replacement policy, enabling informed decisions and optimizing long-term efficiency. An MDP for a herd of dairy cows without interaction between animals is proposed. The model is an adaptation for dairy cows of the models presented by Plà-Aragonés [19,20]. The transition times of the model are monthly, and the state variables considered are the month of lactation (MIL), month of pregnancy (PREG), and number of parity (PAR). Months in lactation can take values from 1 ≤ MIL ≤ 12, so that the maximum number of months a cow is milked is fixed at 12 months. Parity covers the range of 0 ≤ PREG ≤ 9, being PREG = 0 when the cow is not pregnant, and PREG ≥ 0 when it is the *i*-month of gestation. When PREG = 9, calving occurs. The maximum parity considered is PAR = 15, and then parity can take values from 1 ≤ PAR ≤ 15, with the cow starting in the model at PAR = 1 and being replaced whenever PAR = 15 is completed. At the beginning of the process, all cows are in the state MIL = 1, PREG = 0, and PAR = 1. At each monthly time step, the state of each cow updates its values of MIL, PREG, and PAR according to the actual state of the cow and feasible transition probabilities like pregnancy, abortion, death, and involuntary culling. These probabilities depend on MIL and whether the cows are primiparous or multiparous. The monthly transitions of the model can be represented by a directed graph as shown in Figure 1, where feasible transitions from one state to the next one (1, PREG, MIL) are represented by direct arcs of each month in milk and pregnancy at the first parity, and when a cow goes to parity 2, it repeats the same structure. A cow is inseminated when it is not pregnant (PREG = 0). It is after one month, depending on the failure or success in conception, that the cow can remain non-pregnant (PREG = 0) or move to the first month of pregnancy (PREG = 1). The conception or pregnancy rate is generated through a binomial distribution. A milking pregnant cow increases the MIL level until calving (PREG = 9), then moves to the next parity. In the progression towards PREG = 9, the cow may interrupt the regular path to calving due to two possible causes: (i) the occurrence of an abortion by which the cow remains lactating progressing to the level MIL + 1 and PREG = 0 maintaining the same level of parity, (this rule will be changed when the original model is modified to introduce abortions in greater detail), and (ii) death or involuntary culling through a culling rate. When a cow is culled, it is immediately replaced by a new cow with the initial state: MIL = 1, PREG = 0, and PAR = 1. A cow at the initial state will wait two months to be inseminated, starting a reproductive program. In this way, in lactation month 3, the cow can be pregnant (MIL = 3, PREG = 1) or not pregnant (MIL = 3, PREG = 0). In this way, the original model characterizes a cow according to parity, pregnancy, and month in lactation according to the coordinates (PAR, PREG, MIL). It is important to note that not all transitions between two (PAR, PREG, MIL) states are allowed (see Figure 1). For example, it is not possible to reach the states MIL = 1, PREG = 1 from any parity, nor the state MIL = 3, PREG = 2 from any parity. Each parity has 24 × 10 = 240 possible states, but by removing the 54 states that are not possible, the feasible states per parity are reduced to 186. So, when considering the 15 parity levels, each cow can be in only one of 186 × 15 = 2790 feasible states. Whilst the transition matrix order is 3600 × 3600, since it contains all the transition probabilities that include the feasible and not feasible (24 × 10 × 15).

To validate the implementation of the base model, we are going to compare it with the steady-state results presented by Cabrera [10]. These results correspond to the replacement policy of slaughtering non-pregnant primiparous cows at MIL = 11 and slaughter multiparous cows at MIL = 10. The comparisons of herd distributions involve (i) the distribution of cows over MIL, (ii) the distribution of cows over PAR, and (iii) the joint distribution of MIL and PREG.

**Figure 2:**
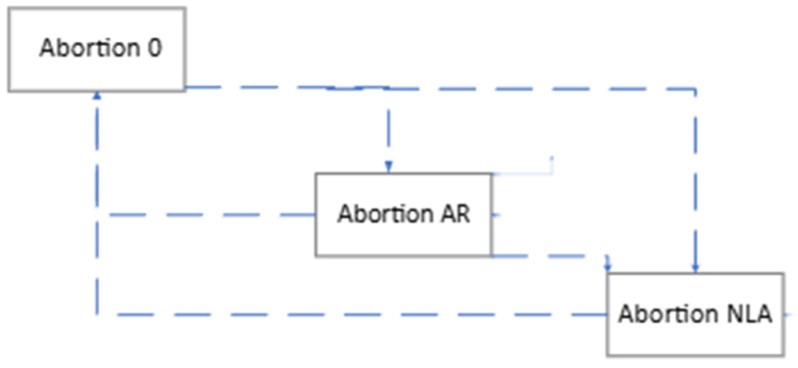
Simplified representation by blocks of feasible transitions of cows regarding abortion: no abortion or AB = 0, RA or AB = 1, and NLA or AB = 2.

The previous model is extended to consider specific characteristics of abortions as presented by Keshavarzi et al. [16]. For this reason, a fourth dimension that represents the number of abortions (AB) a cow has suffered is added to the state variables. Values for AB range from zero to two, being zero for a cow that has never aborted, one for a cow that has suffered an RA, and two for a cow that has suffered an NLA. Thus, the extended model considers that each cow state is characterized by the coordinates of abortion, parity, pregnancy, and lactation (AB, PAR, PREG, MIL). The hierarchic digraph representing feasible transitions in the abortion dimension is shown in Figure 2, where three blocks are shown, each grouping the states with the same abortion value for a cow, and encapsulating the model represented by Figure 1. In particular, an abortion before 60 days of gestation (PREG ≤ 2 months) then it will not be considered as such, and therefore, the cow will remain at the same abortion type level. On the other hand, if gestation is over 60 days and less than 260 days (2 < PREG ≤ 9), cows are likely to experience an RA 1–1.9/12.5 ≈ 84.8% [16] moving to Block AB = 1, or an NLA with a probability of 1.9/12.5 ≈ 15.2% [16] going to Block AB = 2. Always, an abortion provokes a cow to move to PREG = 0, but if the abortion is RA, the cow is milking and progress to MIL + 1 within the same parity, while if the abortion is NLA the cow is not milking and so MIL = 1 and increase its parity by one (PAR + 1). Otherwise, when a cow is culled regardless of the AB-value, then the new cow starts in Block AB = 0 with state values of (0, 1, 0, 1). The unfeasible states will have the same PAR, PREG, and MIL values in each of the AB states from zero to two. In this way, the new transition matrix will have the dimensions of 10,800 rows and 10,800 columns.

Given the complexity of the original model and especially the extended model that considers different states of abortion, a code in Python v 3.13 [21] was written. As a programming language widely used in research, it also facilitates sharing and reproducing the work with other researchers.

We consider the probabilities of pregnancy and death and involuntary culling proposed by Cabrera [10] as summarized in Table 1. Transition probabilities depend on the month of lactation and whether the cow is primiparous or multiparous. Probabilities of abortion are borrowed from De Vries [22], being 3.5%, 2.5%, 1.5%, 0.5%, 0.25%, 0.1% and 0.1% for months of gestation 2 to 8, and therefore the probability of abortion is 8.45%. The first month of gestation is considered with a probability of abortion of zero because it is considered that in that case the abortion is not detectable.

The productive and reproductive impact of cows suffering NLA and RA abortions [16] is presented in Table 2.

The present value of a cow is calculated by the difference between total income minus total cost, multiplied by the probability of the steady state c, as shown:

$${NPV}_{cow}=\underset{c}{\sum }{NPV}_{c}*\;{Prob}_{c}=\underset{c}{\sum }\left({Income}_{c}-{Cost}_{c}\right)*{Prob}_{c}$$

Incomes and costs considered are as follows:

$$\left({Income}_{c}-{Cost}_{c}\right)=({IOFC}_{c}+{INB}_{c}+{VD}_{c})-(VRc\mp \;{CIC}_{c}\mp \;{Al}_{c}\mp VCc\mp PGc)\;$$

where *IOFC* is milk income over the feed cost, *INB* the input of newborn calf (calf value), *VD* is the salvage value of a replaced cow (carcass value), *VR* the replacement cost (replacement heifer cost), *AI* the cost of artificial insemination, *VC* is the veterinary cost, and PG is the loss by twin farrowing. The *IOFC* value will be calculated as follows:

$${IOFC}_{c}={Mp}_{c}*{MP}_{c}-{DMI}_{c}*FC$$

where *Mp* is the monthly milk production, *MP* is the price per liter of milk, DMI is the dry matter intake, and FC is the feed cost per kg of dry matter [23], values available in Table 3.

Added to these data are the considerations that DMI = 25 kg/day [24] for each cow and that the monthly milk production level for each cow according to its parity is obtained from the data provided by Angel Vásquez et al. [25] and represented in Table 4.

To perform the optimization, the available Python libraries were used, such as scipy. optimize.

## 3. Results

**Figure 3:**
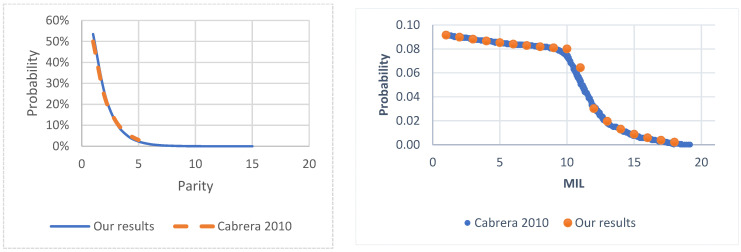
**Left**: Representation of the probability of states as a function of parity according to the results of our model and those obtained by the analytical model. **Right**: Herd structure for the different levels of month in lactation obtained by us (points) and the one presented by Cabrera [10] (continuous line).

To validate our model, we first examine the herd structure at the steady state of the base model (Figure 1) without including the characteristics of RA or NLA types of abortions (equivalent to keep AB = 0 as constant) for the selected specific case of slaughtering open cows in second parity at MIL = 11 and subsequent parities at MIL = 10. We obtain the parity distribution in the left panel of Figure 3, the MIL distribution in the right panel of Figure 3, and the detail of the PREG and MIL distribution for the second parity in Table 5.

After the validation, we solve the extended model considering AB = 0, 1, or 3, and we compare the outcome of both models. In Table 6 it is shown the percentage difference between our results and those presented by Cabrera [10] with the detail of the structure in second parity of the steady state when the slaughter of non-pregnant cows is performed at lactation month 11 for the first parity (MIL = 11, PREG = 0 and PAR = 1) and lactation month is 10 for the following parities (MIL = 10, PREG = 0 and PAR > 1). The deviations in Table 6 with our model are less than 1%.

**Figure 4:**
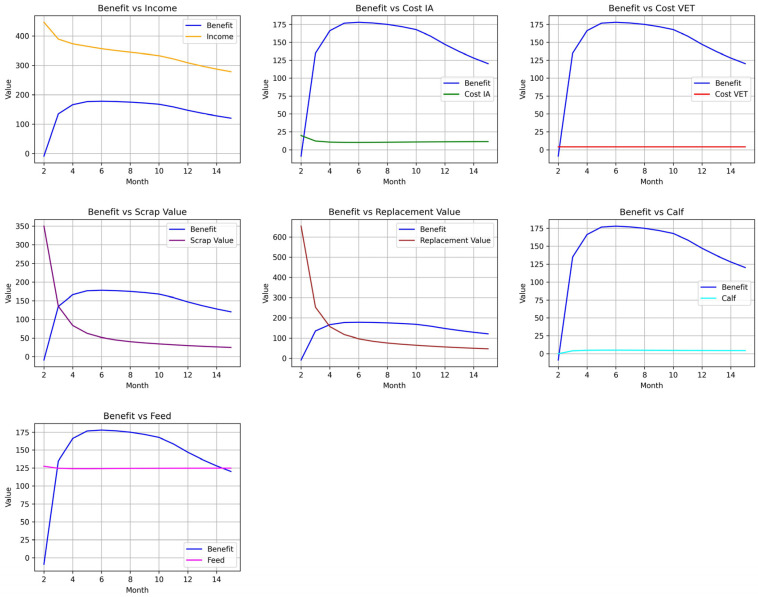
Comparison between the net benefit of each cow and income, cost of artificial insemination, cost of veterinary services, slaughter value, replacement value, value of calves born, and feeding for different months of voluntary culling of open cows.

The results of the extended model that considers RA and NLA according to the new data parameters summarized in Table 2, show the impact on production and reproduction performance of abortions. In the first instance, we run the model performing the culling for non-pregnant cows (i.e., open cows) for different values of MIL and taking into account the impact of reduced milk production and increased probability of involuntary slaughtering of cows that have experienced abortion compared to those with normal calving, data. In this way, we obtain the benefit per animal per month for each of the MIL levels for open cows where voluntary culling is carried out, values presented in Table 7. We can see that the system reaches the maximum benefit of USD 178.77 when the policy of culling open cows that reach the sixth month of lactation failing to become pregnant in the case of primiparous cows. For comparison purposes, Table 7 also shows the result obtained with the base model that does not consider the characteristics of RA and NLA abortions (i.e., the animals are always kept in AB= 0 as shown in Figure 2) again following the Markovian decision model methodology, also obtaining the optimal benefit of USD 178.08 for open cows when they are culled at MIL = 6 for primiparous cows. The net benefit of each level of slaughter depends on the calculation of income minus costs, which is used to identify the optimum. The income, cost of artificial insemination, veterinary costs, waste value of the slaughtered cows is reviewed, replacement cost, income per calves born and the feeding costs of each level of voluntary culling and their results are presented in Figure 4.

Regarding the optimal solution, the steady state where voluntary culling for open cows is performed at the lactation month level of MIL = 6, the total relative number of cows that have experienced an abortion is 2.96%, a figure that is consistent with the probabilities of abortion presented in the Materials and Methods section. Considering the above abortion percentage, we note that as expected cows experiencing an RA abortion is much higher (2.37%) than for cows with NLA abortion (0.59%), which is due to the fact that the latter are less frequent and stays more month in milk (MIL) than cows that experience abortions in earlier stages of lactation. This is reinforced by Figure 3 where it is shown that the percentage of animals in the steady state that are in higher MIL stages is lower compared to smaller MIL values, therefore its occurrence is less likely [10,16]. The modified model and the original model have shown very similar herd structures, which can also explain why, despite the fact that different voluntary culling values are considered, the probabilities of abortion are relatively low and do not exceed 3% for any level of pregnancy month. Finally, when we analyze the results of Table 7, we see that when cows experience abortions there are an increment in days of non-pregnancy and implies changes in the probabilities of non-pregnancy. When we simulate different values of this change in probability from 2% to 60% of non-pregnancy we observe a decrease in net benefit of USD 1.59 per cow per month, which may be explained because herd-level abortions are relatively rare events due to their low probability of occurrence.

## 4. Discussion

There are studies claiming that there is a lack of comprehensive information on the economic implications of culling decisions [26] and proposing replacement models involving stochastic and dynamic characteristics to evaluate these decisions [6]. The dairy cow replacement model that we have described is an extension to methodologically similar models previously presented by other authors [8,10,14] and even for other species [19,20], where we have added characteristics of RA and NLA. Although it is known that abortions impact on the production and reproduction of cows, this impact has not yet been evaluated economically. Vindas-van der Wielen et al. [17] recently conducted an observational study evaluating the impact of two types of abortions classified in early fetal mortality (like our RA) and late fetal mortality (equivalent to our NLA). However, the study was limited to tropical productive conditions, and the results are not comparable to ours.

Most studies opt for simulation methods more flexible than optimization models [6,13,23,26,27,28,29]. These models are good to evaluate management strategies and culling decisions one by one [29], but not so for identifying the best one. However, many of the published replacement models have inspired decision tools [12,14,30,31], some of them based on spreadsheets like Groendal et al. [30] or Cabrera [14]. However, in this paper, we have selected a programming language considering the advantages of scientific programming in Python [31]. We have written our own code that, in the future, will allow us to modify the model with greater flexibility. This way, we will be able to evaluate the economic impact of variations in productive and reproductive variables of the herd or perform a sensitivity analysis on the main input parameters, which can translate into better decision-making tools for farmers. Other potential uses of this kind of model, derived from the information they can provide, are educational use as proposed by Casamiglia et al. [32].

Our extended model demonstrates the impact of abortions at the beginning of the pregnancy (RA) or at a later stage (NLA), which corresponds to the decrease in milk yield, i.e., income, and the increase in the culling rates of these cows, i.e., cost. However, the economic impact of abortions is only one aspect among the many others that we can explore with this replacement model [1,11]. However, most of the replacement models have considered the risk of abortion just as input, or they have not considered it at all [27]. Reasons for that could be the difficulty of having good registries of abortion events, provoking some observational studies to explicitly ignore these kinds of records [33].

The comparison between the base model without abortions (as shown in Figure 1) and the model of Cabrera [10] has served to validate our proposal: it represents a strong basis to verify and validate analytically our approach, which has shown coherence with previous results. In this way, trust in later extensions of the model requiring much larger transition matrices is generated, as we have done, including abortions. In this way it is possible to argue with greater emphasis that the differences between the model that considers abortions and those that do not are explained due to the new features introduced in the model and not to implementation errors. This also provides the reader with a comparative basis for the results of the modified model.

Figure 3 shows the steady-state herd structure of our base model, presenting close results to those of Cabrera [10] for the percentages of PAR and MIL. Similarly, Table 5 shows the details of our results from the original model for the different states of PREG and MIL when the second parity is considered. The relative differences between the results of Table 5 and the corresponding results of Cabrera [10] are found in Table 6, from which we can highlight that, in general, these differences are less than 1% in each state presented. This suggests that our model is coherent and provides a great opportunity to implement new characteristics to better understand the dynamics of the productive and reproductive performance of dairy cows.

In the extended model with abortions, we have observed that the benefit is maximum in the herd if non-pregnant primiparous cows are culled at six months of lactation, the average net value per cow per year was USD 2145, equivalent to USD 178.77 per cow per month. The results of Giordano et al. [23] showed a net value per cow of USD 3179 for the case of the artificial insemination program with pregnancy rates for the first service of 42% and 30% for subsequent services based on a Markov decision process formulation considering daily transition probabilities for the events of aging, replacement, mortality, pregnancy, and calving. The difference with our proposal is mainly due to the day-to-day representation of the herd and the optimization algorithm. Two years earlier, Cabrera [10] calculated the structure and economic value of a dairy herd also employing a Markov decision processes model using monthly transition probabilities between states, and different types of feeding diets for cows, the associated costs of the diet for nitrogen excretion were considered and included in the net value calculated per cow per month ranging between USD 10.98 and USD 132.16, having in this case lower values than those obtained by our model. This difference with respect to our proposal is mainly due to the use of different input values than those presented by Giordano et al. [27], in addition to the consideration of different types of diets and environmental costs associated with nitrogen excretion. Similarly, Table 7 shows that the model that does not take into account the special characteristics of abortions also obtains the maximum benefit for voluntary culling for open cows at MIL = 6, differing only by USD 0.69 per cow per month. This brings us back to the idea that the use of Markov chains for this type of problem implies that a small change that consists of incorporating new details into the herd model requires a greater effort in the implementation of the new transition matrix [9], it would possibly be necessary in the future to explore simulation models to add more features with possibly less effort [7]. The study of Figure 4 indicates that for levels of 1 ≤ MIL ≤ 6 for slaughter, the net value increases mainly due to the fact that the magnitudes of the slopes of slaugther values and replacement value are more pronounced than that of revenue, all of them being negative, which leads to a decrease in revenue by less than the sum of slaugther value and replacemet value and therefore causes an increase in net profit as MIL increases. For lactation levels greater than six, it is observed that the slope of income is approximately maintained, while the slopes of slaughter values and replacement value become more and more similar to zero, so the situation is reversed with respect to the other tranche, and therefore, the net profit decreases as MIL increases.

As indicated by Keshavarzi [16], one of the characteristics of RA and NLA abortions that we have not considered in the model is the fact that the days of non-pregnancy in RA abortions increase by 132 days, while the days of non-pregnancy in NLA abortions decrease by 15 days, which added to the fact that AR abortions have a higher probability of occurrence suggests that the net benefits calculated in this work are to some extent overestimated. However, we confirm that introducing this feature to the model implies increasing its complexity as the order of the transition matrix considerably increases [1]. On the other hand, the introduction of these features in a simulation model would be relatively simpler [34].

The model was designed to be applicable in real contexts of dairy production. Although its mathematical structure is complex, the results it provides, such as the monthly economic benefit per cow according to different culling decisions, are easy to interpret [1]. This allows veterinarians and producers to make informed decisions without the need for advanced technical knowledge. In addition, as it is implemented in Python, the model can be integrated into decision support systems (DSSs), facilitating its practical use. Yousaf et al. [35] remark that DSSs based on programming tools and agriculture 4.0 are being implemented to optimize operations in agriculture

## 5. Conclusions

We have generated an analytical model of dairy cow herds that contemplates characteristics such as voluntary replacements, involuntary replacements, mortality, pregnancy through artificial insemination and abortion events according to their type: rebreeding abortion (RA) where the cow maintains its lactation level and new lactation abortion (NLA) where cows experience these events and go to the next lactation state. Using Markov decision processes and writing the transition matrix code in Python and the solution to find the stable states, we proceeded to perform the economic evaluation of the system. In our validation process, our model was able to accurately reproduce the results found in the literature. When we examine our extended model that includes the RA and NLA abortions, we notice that the optimal situation occurs when open cows are in lactation month six for primiparous cows. Our net benefit results are within the range indicated by the literature. The model that considers the types of abortions has a difference of USD 0.69 per cow per month of net benefit with the case of not considering the types of abortions. Our problem consisted of solving the problem of optimization of the voluntary culls of open cows, considering in this case the NLA and RA abortions, which implies adding a new characteristic to the model, but resulting in a greater complexity with larger transition matrices. So, solving this kind of problem using exact methods poses a great challenge if more characteristics are to be considered. For future work, we propose to investigate the stochastic simulation method to obtain insight into more complex problems, which include a great number of characteristics, providing a good choice for the future.

## Figures and Tables

**Table 1:** Summary of pregnancy rate, mortality, and involuntary culling [10].

	Pregnancy Rate		Primiparous		Multiparous	
MIL	Primiparous	Multiparous	Mortality	Involuntary Culling	Mortality	Involuntary Culling
1	0	0	0.004	0.006	0.012	0.018
2	0.2368	0.2103	0.004	0.006	0.011	0.017
3	0.1888	0.1795	0.004	0.005	0.011	0.016
4	0.1363	0.1429	0.003	0.005	0.010	0.015
5	0.0995	0.1107	0.003	0.005	0.010	0.014
6	0.0748	0.0875	0.003	0.004	0.009	0.013
7	0.0573	0.068	0.003	0.004	0.009	0.011
8	0.0449	0.0535	0.003	0.003	0.008	0.010
9	0.0428	0.0527	0.003	0.003	0.008	0.009
10	0.0458	0.0546	0.002	0.003	0.007	0.008
11	0.0493	0.0516	0.002	0.002	0.007	0.007
12	0.0498	0.0541	0.002	0.002	0.006	0.006
13	0.0517	0.0557	0.002	0.002	0.006	0.006
14	0.0505	0.0575	0.002	0.002	0.006	0.007
15	0.0501	0.0549	0.002	0.002	0.006	0.007
16	0	0	0.002	0.002	0.007	0.007
17	0	0	0.002	0.002	0.007	0.007
18	0	0	0.002	0.003	0.007	0.008
19	0	0	0.002	0.003	0.007	0.008
20	0	0	0.002	0.003	0.007	0.008
21	0	0	0.002	0.003	0.007	0.008
22	0	0	0.002	0.003	0.007	0.009
23	0	0	0.002	0.003	0.007	0.009
24	0	0	0.0025	0.003	0.008	0.009

**Table 2:** Productive and reproductive impact on cows experiencing NLA and RA abortions reported by Keshavarzi et al. [16].

	NLA	RA
Reduction in milk production yield	19.4%	7.3%
Increased risk of culling compared to cows with normal calving (times)	1.9	
Increased non-pregnancy days compared to normal calving	−15	+132

**Table 3:** Economic parameters of the model [23].

Price	Value
Milk (USD/kg)	0.36
Calf value (USD/calf)	100
Carcass value (USD/kg)	1.16
Replacement heifer cost (USD)	1.300
Veterinary cost (USD)	50
Feed cost in lactation (USD/kg)	0.17
Feed cost in dry period (USD/kg)	0.13

**Table 4:** Monthly milk production kg/month [25].

Month	2nd Parity	3rd ≤ Parity
1	1252	1025
2	1228	1333
3	1187	1332
4	1129	1264
5	1103	1181
6	935	1025
7	969	1076
8	868	920
9	827	858
10	703	719

**Table 5:** Detail of steady-state structure for the second parity, base model, when the culling of open cows is 11 for first parity and 10 for subsequent parities.

	PREG								
MIL	0	1	2	3	4	5	6	7	8
1	0.024984								
2	0.024243								
3	0.018609	0.004956							
4	0.014866	0.003252	0.004825						
5	0.012592	0.002072	0.003172	0.004541					
6	0.011161	0.001362	0.002024	0.002990	0.004325				
7	0.010173	0.000956	0.001333	0.001911	0.002853	0.004169			
8	0.009429	0.000678	0.000937	0.001260	0.001826	0.002754	0.004065		
9	0.008853	0.000495	0.000666	0.000887	0.001207	0.001766	0.002690	0.003982	
10	0.008310	0.000459	0.000487	0.000632	0.000851	0.001169	0.001728	0.002639	0.003912
11			0.000452	0.000463	0.000607	0.000826	0.001146	0.001698	0.002597
12				0.000430	0.000445	0.000590	0.000811	0.001127	0.001674
13					0.000415	0.000434	0.000580	0.000799	0.001113
14						0.000404	0.000426	0.000572	0.000789
15							0.000397	0.000420	0.000564
16								0.000391	0.000414
17									0.000385

**Table 6:** Relative differences between our results and those presented in Cabrera [10] detailing steady-state structure in the second parity.

	PREG								
MIL	0	1	2	3	4	5	6	7	8
1	5%								
2	4%								
3	4%	3%							
4	4%	2%	3%						
5	3%	3%	2%	1%					
6	2%	5%	1%	3%	1%				
7	2%	6%	2%	1%	2%	2%			
8	0%	−3%	4%	−3%	1%	2%	−1%		
9	−1%	−1%	−5%	−1%	1%	−2%	0%	0%	
10		−9%	−3%	5%	−6%	−3%	2%	−2%	−2%
11			−11%	−8%	1%	3%	−5%	0%	0%
12				7%	−12%	−2%	1%	−6%	−2%
13					4%	8%	−3%	0%	1%
14						1%	6%	−5%	−1%
15							−1%	5%	−6%
16								−2%	3%
17									−4%

**Table 7:** Average net benefit per cow obtained by the modified abortion model and the original model. The final column represents the absolute difference between the two models.

	Profit per Cow		
Month of Culling	Model with RA and NLA	Base Model	Difference
2	−8.76	−8.76	0.00
3	134.54	134.77	−0.23
4	166.35	166.20	0.14
5	177.18	176.72	0.46
6	178.77	178.08	0.69
7	177.90	177.13	0.77
8	176.12	175.27	0.85
9	173.14	172.23	0.91
10	169.20	168.17	1.02
11	160.47	159.06	1.41
12	149.16	147.41	1.76
13	139.36	137.30	2.05
14	130.68	128.38	2.30
15	123.03	120.51	2.52

## Data Availability

The original data presented in the study are openly available in [10,16,23,25].
